# Acquisition of biologically relevant gene expression data by Affymetrix microarray analysis of archival formalin-fixed paraffin-embedded tumours

**DOI:** 10.1038/sj.bjc.6604316

**Published:** 2008-04-01

**Authors:** K M Linton, Y Hey, E Saunders, M Jeziorska, J Denton, C L Wilson, R Swindell, S Dibben, C J Miller, S D Pepper, J A Radford, A J Freemont

**Affiliations:** 1 Cancer Research UK Department of Medical Oncology, Christie Hospital NHS Foundation Trust, Wilmslow Road, Withington, Manchester, M20 4BX, UK; 2 School of Clinical and Laboratory Sciences, The University of Manchester, Oxford Road, Manchester, M13 9PT, UK; 3 Cancer Research UK Paterson Institute for Cancer Research, The University of Manchester, Wilmslow Road, Withington, Manchester, M20 4BX, UK; 4 Almac Diagnostics, Seagoe Industrial Estate, Craigavon, BT63 5QD, Northern Ireland; 5 School of Cancer and Imaging Sciences, The University of Manchester, Oxford Road, Manchester, M13 9PT, UK

**Keywords:** archival formalin-fixed paraffin-embedded tissue, gene expression profiling, Affymetrix U133 Plus 2.0 array, extremity soft tissue sarcoma, prognostic markers

## Abstract

Robust protocols for microarray gene expression profiling of archival formalin-fixed paraffin-embedded tissue (FFPET) are needed to facilitate research when availability of fresh-frozen tissue is limited. Recent reports attest to the feasibility of this approach, but the clinical value of these data is poorly understood. We employed state-of-the-art RNA extraction and Affymetrix microarray technology to examine 34 archival FFPET primary extremity soft tissue sarcomas. Nineteen arrays met stringent QC criteria and were used to model prognostic signatures for metastatic recurrence. Arrays from two paired frozen and FFPET samples were compared: although FFPET sensitivity was low (∼50%), high specificity (95%) and positive predictive value (92%) suggest that transcript detection is reliable. Good agreement between arrays and real time (RT)–PCR was confirmed, especially for abundant transcripts, and RT–PCR validated the regulation pattern for 19 of 24 candidate genes (overall *R*^2^=0.4662). RT–PCR and immunohistochemistry on independent cases validated prognostic significance for several genes including *RECQL4, FRRS1, CFH* and *MET* – whose combined expression carried greater prognostic value than tumour grade – and cmet and TRKB proteins. These molecules warrant further evaluation in larger series. Reliable clinically relevant data can be obtained from archival FFPET, but protocol amendments are needed to improve the sensitivity and broad application of this approach.

Whole-genome microarray gene expression profiling has rapidly become the gold standard platform for retrospective prognostic and predictive gene discovery in human cancers, but currently depends on fresh-frozen tissue (FT) as a source of high quality RNA. The majority of archival tumour specimens exist as routinely prepared formalin-fixed and paraffin-embedded tissue (FFPET), which is generally regarded as being unsuitable for microarray applications due to the presence of degraded and chemically modified RNA. Whereas RT–PCR interrogation of archival FFPET has proven to be a reliable tool and is widely used (Cronin *et al*, 2004), this approach is unsuitable for large scale discovery-based investigation owing to the limited number of genes that can be assayed in one experiment. RNA from freshly prepared FFPET may be minimally degraded (von Ahlfen *et al*, 2007) and their transcript profiles correlate very well with those from paired unfixed FT (Lee *et al*, 2005a; Scicchitano *et al*, 2006; Frank *et al*, 2007), but these samples lack the requisite clinical follow-up data for correlation with expression data.

RNA degradation *per se* does not preclude microarray analysis with Affymetrix platforms (Schoor *et al*, 2003; Lee *et al*, 2005b), which by design are ideally suited to interrogate RNA fragments. Chemical modifications on the other hand not only hinder RNA extraction but also impede reverse transcription and amplification reactions (Godfrey *et al*, 2000; Lee *et al*, 2005b). The main concern with using routinely prepared/archival FFPET for microarray profiling is that ‘block effects’, such as pre-fixation time, size of specimen being fixed, formalin/tissue processing conditions and length of time in storage, will have variable effects on RNA degradation and modification rates and consequently adversely affect the reliability and clinical interpretation of microarray data. The impact of ‘block effects’ on RT–PCR and microarray performance is only just coming to light (Penland *et al*, 2007; von Ahlfen *et al*, 2007), but may not be as problematic as feared especially considering that RT–PCR (the gold standard assay for FFPET interrogation) has demonstrated reliable performance despite the presence of these factors. This may also be true for microarray applications. In a study of 157 archival FFPET samples aged 2–8 years, correct classification of tumour type and subtype using an unsupervised approach suggests that biological data are more powerful than ‘block effects’ (Penland *et al*, 2007). Furthermore, several groups have compared FT and FFPET arrays in paired and unpaired samples and have shown that archival FFPET specimens can retain valuable and reliable transcript data. For example, using Illumina manufactured cancer-specific oligonucleotide bead microarrays and DASL amplification of hybridised templates, Bibikova *et al* found similar transcript profiles in routinely processed archival FFPET and paired FT specimens, although fewer genes were detected in FFPET (Bibikova *et al*, 2004), while Haque *et al* used FFPET arrays to independently validate gene expression profiles obtained from FT samples of paediatric glioblastoma (Haque *et al*, 2007). More recently, Bibikova *et al* were able to show that archival FFPET expression signatures correlate with Gleason score in relapsed prostate cancer (Bibikova *et al*, 2007), attesting to the clinical and prognostic value of FFPET transcripts.

It is apparent that highly concordant qualitative data can be obtained for genes called ‘present’ in paired archival FFPET and FT 22K oligonucleotide arrays, especially for better quality FFPET (as evidenced by the presence of ribosomal peaks) (Coudry *et al*, 2007), but the reproducibility of quantitative data (i.e. relative gene expression fold changes) and degree of concordance when nonexpressed (‘absent’) genes are taken into account is poorly understood.

These studies raise an important question: can FFPET profiles be used *de novo* to generate valid quantitative prognostic data? The primary objectives of our study were first, to test whether microarray profiling of archival FFPET can provide similar quantitative data to those obtainable from RT–PCR and second, to determine whether these data have clinical/prognostic relevance. Confirmation of these aims would support the notion of using archival FFPET profiling for biomarker discovery in tumours where FT is in short supply. We chose to study extremity soft tissue sarcoma (STS) as an example of a rare tumour where the vast majority of tissues exist only as FFPET. A secondary aim was to identify promising prognostic biomarkers in this disease entity.

## MATERIALS AND METHODS

### Study population

Cases of completely resected, localised, extremity STS (leiomyosarcoma, liposarcoma and synovial sarcoma) were retrospectively identified from Christie Hospital and Manchester University Medical School Records. Pathological and clinical follow-up data were collected for all patients. Two prospectively identified cases (a leiomyosarcoma and spindle sarcoma not otherwise specified) were included for assessment of paired FFPET and FT. Twelve benign tumours (seven lipomas and five leiomyomas) were collected from University archives for comparison of gene expression with their malignant counterparts.

### Preparation of tissue samples

Tissues were used in accordance with multi-centre research ethics committee guidance and with informed patient consent. FFPET samples were retrieved from local pathology departments where they had been routinely processed and stored for 1–8 years (mean 6 years). Ten-micron thick sections were cut from representative tissue blocks. After discarding the top few sections (to eliminate oxidised/contaminated tissue), viable tumour and adjacent stromal tissue areas were carefully macrodissected from tissue sections using a scalpel and dissecting microscope. Care was taken to avoid contamination by exogenous RNases and sample cross contamination by changing gloves frequently, decontaminating all surfaces and equipment with RNase eliminating solutions and cleaning with xylene between samples to eliminate wax carryover.

FT samples were collected in the operating theatre, divided into 1.0 cm^2^ pieces and immediately placed in TRIzol reagent (Invitrogen, CA, USA) prior to freezing at −80°C.

### RNA extraction

Total RNA was isolated from FFPET using the Optimum FFPE extraction protocol (Ambion Diagnostics, TX, USA), with minor modifications, including incubation with a further 300 units of proteinase K at 50°C for 2–4 h for samples with residual undigested tissue as this gave significantly higher purity and yields of total RNA (data not shown). RNA was extracted from thawed, homogenised FT using the TRIzol method, according to manufacturer's instructions. All RNA samples were DNase-treated (Optimum Kit) and purified (RNeasy Micro Kit, Qiagen, Hilden, Germany). Total RNA yield and purity were estimated by ultraviolet spectroscopy (Nanodrop ND-1000 Spectrophotometer, Nanodrop Technologies, DE, USA) and quality was assessed on an Agilent 2100 Bioanalyzer (Agilent Technologies, CA, USA).

### Affymetrix expression microarrays

Thirty-four FFPET samples were selected for microarray experiments based on total RNA yield >2 *μg* and 260 of 230 ultraviolet absorbance ⩾1.8. Two micrograms of total RNA were used to prepare biotinylated target RNA using the Affymetrix One Cycle Target Preparation Protocol driven by T7-linked oligo(dT) primers. Manufacturer's recommendations were followed, apart from complementary RNA fragmentation, which was shortened to 15 min. Samples were hybridised overnight to Affymetrix HG U133 Plus 2.0 arrays, scanned and processed using GeneChip Operating Software. Analyses were performed using BioConductor (Gentleman *et al*, 2004). RMA and MAS5 data were produced using the implementations found in the ‘affy’ (Gautier *et al*, 2004) and ‘simpleaffy’ (Wilson and Miller, 2005) BioConductor packages. MAS5 expression calls were generated using the simpleaffy implementation of (Hubbell *et al*, 2002) and detection calls with the simpleaffy implementation of (Liu *et al*, 2002). Unless otherwise stated, *α*1 and *α*2 values were 0.05 and 0.065 respectively, the default values for these arrays. All MAS5 data were scaled to a target intensity of 100.

### Selection of microarray training samples based on Affymetrix QC parameters

**Figure 1:**
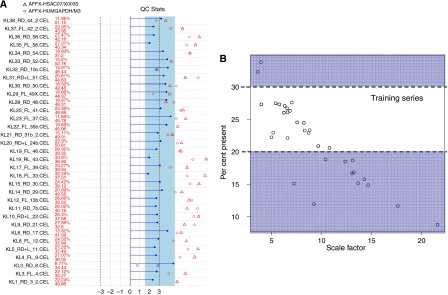
(**A**) Affymetrix quality control parameters for all FFPET arrays: displayed in the first column, present calls (top) are highly variable (from 8.8 to 33.8%) while background (bottom) is fairly constant across the dataset. Scale factors should be within threefold of each other for arrays to be comparable; range of scale factors (horizontal lines) is 3.64–21.61, with many falling outside the three-fold dotted lines. GAPDH and ß-actin 3′5′ ratios (circles and dots) are extremely high and effectively indicate that the 5′ probe set is absent – even the 3′ mid ratios are too high and fall outside Affymetrix recommendations. (**B**) Percent present and scale factors for selected training FFPET arrays: restricting analysis to those samples with a percent present call greater than 20 but less than 30 (middle ‘training series’ group) gave 19 samples (nine nonrecurrent, 10 metastatic recurrence) with 3343 probe sets called present on all arrays (3292 after eliminating AFFX probe sets). Scale factors are generally within threefold for training samples.

As expected, all FFPET samples contained extensively degraded RNA with a mean RIN of 2.2. (1.2–3.6). There was no statistical difference in the RIN values for training and nontraining sets (*P*=0.51, Mann–Whitney *U* test); training set median RIN 2.3 (2–3.6, *n*=18), nontraining median RIN 2.2 (1.2–2.4, *n*=15). RIN values for FT samples were 3.1 and 5.5. FFPET purity was satisfactory (mean 260/280 of 2.03 and mean 260/230 of 1.70) (Supplementary Table S1). Almost half (15 of 34) of FFPET arrays failed to meet one or more recommended Affymetrix quality control criteria for inclusion in the analysis. The remaining 19 of 34 FFPET arrays – which met the criteria, including scale factors within threefold of each other and present calls ranging from 20–30% – were used as a prognostic training set (Figure 1). These arrays still exhibited significantly lower percent present (PP) calls than is expected from high quality samples and high 3′ of 5′ ratios, in keeping with the poor quality of FFPET RNA samples.

### Microarray data analysis

Several rounds of supervised data analysis were used to identify probe sets upregulated or downregulated in metastatic and nonmetastatic outcome cases. The detection call (Liu *et al*, 2002) was used to identify probe sets with a signal intensity not substantially above background. In the initial round of analysis (Gene list 1), probe sets were eliminated from the analysis unless they were consistently flagged as present (i.e. reliable) in all samples, since it is not possible to distinguish between those flagged absent (i.e. close to background level) due to low gene expression, and those flagged absent due to poor RNA quality. Data were filtered on the basis of *z*-score and fold change to generate the first gene list. A 19-gene signature obtained by K-Nearest Neighbour separation (*k*=3) and leave-one-out cross validation (LOOCV) demonstrated 100% accuracy for classification, however, fold changes were considered to be too small for reliable RT–PCR validation. It was therefore decided to include all probe sets (i.e. those flagged present and absent) in subsequent rounds of analysis.

**Figure 2:**
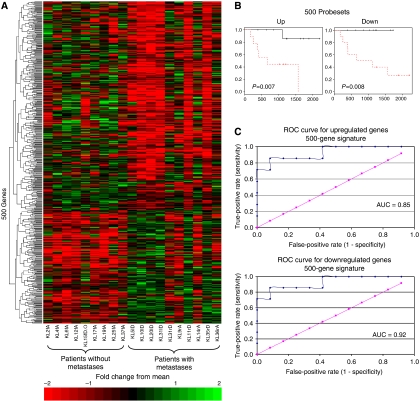
(**A**) Heat map of hierarchical clustering for 500 differentially expressed genes: horizontal rows represent individual genes and vertical rows represent individual patients, grouped by metastatic/no metastatic recurrence. Suffix ‘A’ denotes surviving patients, ‘D’ denotes those who have died of disease, ‘DO’ identifies one patient who did not have recurrent disease at the time of death from nonsarcoma causes. Each cell in the matrix represents the expression level of a single transcript in a single sample, with red and green indicating transcript level above and below the median for that gene across all samples, respectively. It is noteworthy that the expression pattern for the patient who died of nonsarcoma causes shares similarities with those for patients alive without metastases. Highest pattern homology is seen for patients who have died of metastatic disease. (**B**) Log-rank KM plots for up- and downregulated transcripts are shown for samples grouped above/below median gene expression. (**C**) ROC curves and AUC values illustrate sensitivity and specificity of detecting metastatic recurrence within 3 years of diagnosis.

A 500-gene signature was generated using the survival package in R to generate log-odds scores for every probe set on the array (Gene list 2). Functional annotation identified many genes in this list with described roles in cancer biology, suggesting that differential expression was indeed a reflection of underlying biology and not merely borne of statistical chance. The 500-gene list was sorted in order of *P*-value and validated by LOOCV. A series of increasingly less stringent filter cutoffs was used to generate several probe set lists of different lengths. These were used as input to the globaltest package in BioConductor (Goeman *et al*, 2004) to investigate the performance of probe sets when grouped together as a set (rather than treated individually). In this way, a signature comprising the top 50 probe sets was identified (Gene list 3). Changing the filtering parameters to include or exclude probe sets from this list resulted in reduced performance, that is, the 50-gene signature represented the smallest number of genes with collective prognostic power. We used the survival package in *R* (with samples grouped by median expression) to generate Kaplan–Meier (KM) plots for the 19 FFPET arrays to test the relationship between microarray gene expression and survival. This approach is similar to those of (Chi *et al*, 2006; Winter *et al*, 2007). Receiver operating characteristic curves (ROC) for the 500-gene probe sets illustrate the sensitivity and specificity of detecting metastases within 3 years of diagnosis by gene expression, showing good performance with AUC values of 0.85 and 0.92 for up- and downregulated profiles respectively. These data are illustrated in Figure 2 and full gene lists are given in Supplementary Information.

### Candidate gene selection for individual performance validation by RT–PCR

**Figure 3:**
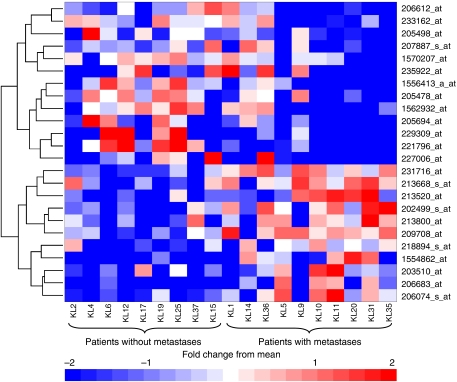
Heat map of hierarchical clustering for 24 selected differentially expressed genes: horizontal rows represent individual genes and vertical rows represent individual patients. Red and blue indicate transcript level above and below the median for that gene across all samples, respectively. Distinct clusters of differentially expressed genes can be seen for patients grouped by metastatic/no metastatic recurrence.

As our primary objective was to test whether quantitative microarray data was reproducible by RT–PCR for archival FFPET, we selected genes from the 500- and 50-gene signatures for individual performance validation by RT–PCR. Twenty-four candidate genes were chosen based on differential fold change between metastatic and nonmetastatic outcome cases, *P*-value, clinical relevance (Table 1) and the ability to design efficient primer pairs for RT–PCR validation. The number correctly classified by LOOCV and associated *P*-value was also used for selecting genes from the 50-gene list. A heatmap depicting the 24 genes is shown in Figure 3.

### Real-time quantitative PCR

Assays were designed using ProbeFinder software (Available at: https://www.roche-applied-science.com/sis/rtpcr/upl /adc.jsp). Where possible, identical Plus 2.0 array targets were interrogated, otherwise the Affymetrix consensus sequence was used. Primers were synthesised by Invitrogen (Paisley, UK) and labelled probes were obtained from the Human Universal Probe Library (Roche, Switzerland). Primer and probe details are given in Supplementary Table S2. CDNA was synthesised using TaqMan Reverse transcription reagents (Reverse-It Kit, ABI, CA, USA) primed with random hexamers, in reaction volumes of 50 *μl* for 1 *μg* total RNA.

Experiments were performed in triplicate on an ABI 7900 Real-Time Sequence Detection System in 384-well format. Assays were tested for efficiency using Human Reference Total RNA (BD Biosciences, CA, USA) and a 10-fold dilution series. Only assays with a slope between −3.00 and −3.60 were carried through. Manufacturer's standard PCR conditions were used and results were analysed using SDS 2.1 (ABI). Mean gene expression *C*_t_ values were normalised to the mean expression of two reference genes (*KIAA0446* [32091_at] and *INTS5* [53968_at] (Supplementary Figure S1). Reference genes were chosen from microarray data for their consistent performance compared with 15 commonly used reference genes and similar stability across RT–PCR assays was confirmed by Genorm analysis (data not shown) (Vandesompele *et al*, 2002).

### Candidate protein selection for performance validation by immunohistochemistry on tissue microarrays

Validation at protein expression level is necessary to fully explore the clinical utility of promising biomarkers, especially as the extent to which mRNA changes are accompanied by similar changes at protein level is highly variable. We were particularly interested in testing known ‘cancer’ genes found to be differentially regulated on FFPET arrays. Dysregulation within the tyrosine ‘kinome’, which includes targets such as c-kit, VEGF, EGFR, is proving to be critically important for tumour development and progression (Bardelli *et al*, 2003). Two receptor tyrosine kinases – *MET* (which encodes cmet) and *NTRK2* (which encodes TRKB) – were found to be highly differentially regulated in our array dataset and, as commercially prepared antibodies were available, they were chosen for protein validation using immunohistochemistry on specially constructed tissue microarrays (TMA).

### TMA construction

Tissue microarrays of extremity STS were constructed in-house using an ATA-100 tissue arrayer (Chemicon International, CA, USA) according to manufacturer's instructions. Each sample was represented by up to four 1.5 mm cores of viable tumour tissue, to allow inclusion of heterogeneous tumour areas and to minimise data loss occurring as a result of core loss or damage during experimentation. TMA sections of 5 *μm*-thickness were cut and processed for immunohistochemistry experiments according to standard tissue preparation protocols.

### Immunohistochemistry

Polyclonal antibodies were purchased for TRKB (Promega, catalogue no. G1561, WI, USA) and cmet (Zymed, catalogue no. 71–8000, CA, USA). The avidin–biotin complex approach was optimised for each antibody. Briefly, deparaffinised and dehydrated sections were microwaved in citrate buffer, pH 6.0 for antigen retrieval. Nonspecific staining was blocked using CAS block (Zymed Laboratories, CA, USA). Following 4°C overnight incubation with primary antibody, tissues were treated with 2% hydrogen peroxide and then incubated with secondary antibody followed by streptavidin HRP, both for 45 min. The reaction was developed with SG chromogen (Vector Laboratories, CA, USA) and sections were counterstained with Mayer's haematoxylin (BDH Laboratory Supplies, Poole, UK).

### Computerised image analysis of immunostaining

**Figure 4:**
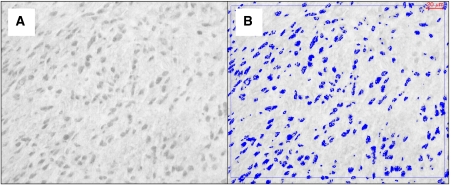
Illustration of cmet immunostaining and computerised image analysis detection of immunostained features in a case of synovial sarcoma. (**A**) Immunopositive areas are dark grey (SG chromogen) and background is light grey, (**B**) computer detected features are marked with blue overlay, which confirms excellent detection of immunopositive areas shown in A.

One digital image per immunostained tissue core was acquired using a Leica RMDB Research Microscope coupled with a DeltaPix camera, and analysed using Quantimet 550 software and an in-house macro written in QUIPS. This enabled automatic computer detection and measurement of immunopositive features (Figure 4). Images were converted to grey scale and each pixel was allocated a value corresponding to its ‘grey shade’. The range of values representing positive staining was previously determined for each antibody by examining staining in positive and negative controls. The sum of all grey values (‘sum grey’) was used to compute quantitative protein expression. For qualitative purposes, samples were considered to have high protein expression if sum grey exceeded the median expression value.

### Statistical analyses

Statistical analyses were carried out in Excel and with SPSS (Statistical Package for Social Sciences) version 10.1. Relative gene expression was computed using the 2^delta–delta *C*_t_ method where delta–delta *C*_t_ is the difference between mean (of triplicate reactions) normalised expression in metastatic recurrence and nonrecurrent cases. Relative protein expression was computed as the ratio between mean sum grey data in metastatic recurrence and nonrecurrent cases. The Mann–Whitney test was used to compare protein expression in benign and malignant tumours and Kruskal–Wallis to compare expression in nonrecurrent, locally recurrent and metastatic recurrent STS. KM analyses were performed using the log-rank test and hazard ratios for metastasis-free survival were generated. AUC values for promising genes were computed. We were also interested in comparing gene expression performance with tumour grade. To do this, we developed a simple scoring system based on gene expression of four promising genes (described in the results section) and used the log-rank test to compare gene score and grade for prediction of metastases within 3 years of diagnosis (see Results).

## RESULTS

### Patient and sample characteristics

Patient and tumour characteristics for 19 FFPET array training cases are summarised in Supplementary Table S3. Median follow-up for surviving patients (*n*=12) was 52.5 months (19–81 months). Ten patients developed distant metastases, with a median time to metastasis of 21 months (2–46 months). At the time of analysis, there were four surviving patients with metastases. One elderly patient without metastases died of nonsarcoma causes.

Patient and tumour characteristics for remaining cases are summarised in Supplementary Table S4. RT–PCR cases (*n*=69) included 15 cases profiled on microarrays but excluded from microarray analysis and 54 independent cases. Immunohistochemistry cases (*n*=85) included 49 of 50 cases also examined by RT–PCR and 32 of 34 cases also profiled by gene microarrays. Median follow-up for surviving patients (*n*=50) was 68 months (19–202 months).

### Comparison of data from paired FFPET and FT RNA samples

**Figure 5:**
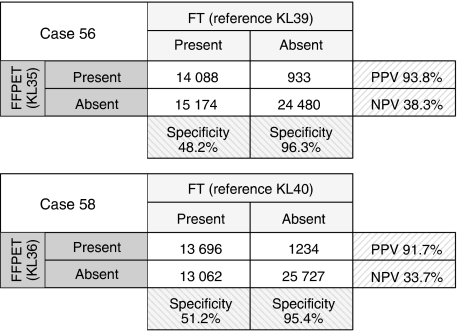
Illustration of sensitivity, specificity, positive predictive values (PPV) and negative predictive values (NPV) for FFPET data relative to paired FT: Two paired FT and FFPET samples were used (cases 56 and 58). Numbers refer to the number of probe sets flagged. TP=true positive, FP=false positive, TN=true negative, FN=false negative. Sensitivity [TP/(TP+FN)] is low, with only ∼50% of transcripts present in FT being detected in FFPET, and corresponding NPV [TN/(TN/FN)] is only 34–38%. Specificity [TN/(FP+TN)] is excellent (95–96%), suggesting that detection calls in FFPET are reliable, with a PPV [(TP/(TP+FP)] of 92–94%.

We related the performance of FFPET arrays to paired FT arrays to test their sensitivity and specificity (sample pairs KL35/KL39 and KL36/KL40). The RIN values for FT samples were unexpectedly low (mean 4.3) but, as mentioned earlier, RNA degradation is not believed to significantly affect the percentage of genes called present on Affymetrix arrays (Schoor *et al*, 2003; Lee *et al*, 2005b). Despite low RIN values, PP rates from the FT arrays were well within the normal range of values seen across all Plus 2.0 arrays run on frozen samples in our laboratory (data not shown). For example, KL39 had RIN 5.5 and PP 48.9% and KL40 had RIN 3.1 and PP 53.5%. This suggests that these FTs were valid reference samples. As expected from previous reports, FFPET array sensitivity was approximately 50% of paired FT (PP 27 *vs* 49% for KL35/39 and 28 *vs* 53% for KL36/40). Only 3–4% of present probe sets were found exclusively in FFPET. This corresponds to high FFPET array specificity (95–96%) and positive predictive value (92–94%), which attest to the reliability of present calls from FFPET arrays (Figure 5). The extent to which FFPET data resembles FT data increased with abundance of expression on arrays (e.g. *R*^2^=0.06 for signal intensities between 5 and 6, compared with *R*^2^=0.49 for signals over 10). Similarly by RT–PCR, genes detected in FT after ∼30 cycles were poorly detected in paired FFPET, while there was generally good agreement for more abundant genes (Supplementary Figure S2). Unfortunately, although these would have been useful, technical replicates were not run for microarrays.

### Comparison of data from array and RT–PCR platforms

**Figure 6:**
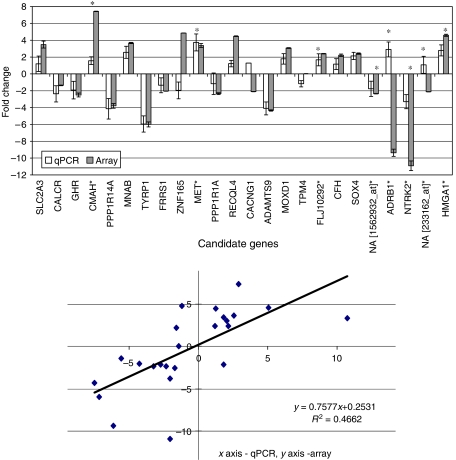
Comparison of paired microarray and RT–PCR assays for candidate genes: genes upregulated in cases with metastatic recurrence (and reciprocally down regulated in nonrecurrent cases) are shown as positive fold changes (above the *x*-axis) and vice versa for negative fold changes. Error bars are the standard error of the difference in the means. Similar upregulated/downregulated fold change patterns were seen for 19 of 24 candidate genes (although magnitudes of change may differ). Agreement was less for eight transcripts where nonidentical targets were probed by microarrays and RT–PCR (identified with^*^).

Real time–PCR is regarded as the ‘gold standard’ assay for validating microarray data (Wang *et al*, 2006), and was therefore used to determine whether microarrays can reproduce the technical ‘truth’ for gene expression. There was good agreement for genes with abundant and consistent expression across the sample series (from which reference genes were selected), as evidenced by Genorm analyses (Supplementary Figure S1). Array data were also generally reproducible by RT–PCR for differentially expressed candidate genes of interest, with similar fold change patterns, although reproducibility was lower when probing for nonidentical sequences (overall *R*^2^=0.4662) (Figure 6). Reproducibility was not affected when different RNA extracts (from the same case) were used for RT–PCR and arrays (data not shown).

### Prognostic value of individual candidate genes and proteins

**Figure 7:**
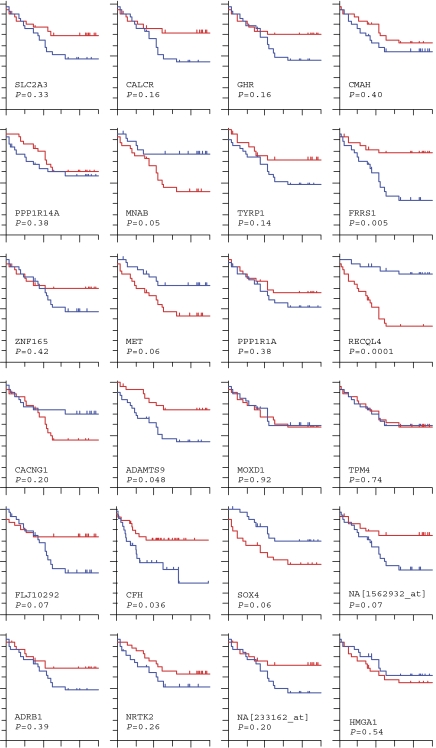
Real time–PCR validation: KM plots for 24 individual candidate genes in 60 samples according to gene expression above/below the median. A statistically significant relationship with MFS was confirmed for five genes (*RECQL4*, *FRRS1*, *CFH*, *ADAMTS9* and *MNAB*), while expression for *MET*, *SOX4*, *NA[1562932_at]* and hypothetical protein *FLJ10292* showed a nonsignificant trend for MFS in this dataset.

The number of FFPET arrays used to generate a prognostic model was small (*n*=19) as almost half of FFPET arrays failed to meet the quality control criteria for inclusion in the analysis. As expected, prognostic value was not seen for all genes when tested across a larger number of samples (Figure 7). Nevertheless, some genes look promising at this early validation stage. Hazard ratios (HR) for candidate genes and proteins significantly associated with survival (*P*<0.05) or associated with a survival trend (*P*<0.1) are given in Supplementary Figure S3. *RECQL4* overexpression increased the risk of metastasis±mortality (log-rank *P*=0.001, HR 5.2 *P*<0.000, while *FRRS1* and *CFH* each reduced metastatic risk (*FRRS1* log-rank *P*=0.005, HR 0.4 *P*=0.017; *CFH* log-rank *P*=0.036, HR 0.44 *P*=0.041. *MET* increased risk, although this did not quite reach statistical significance (log-rank *P*=0.06, HR 2.1 *P*=0.069). At protein level, overexpression of cmet and TRKB each increased metastasis risk (HR 2.3, *P*=0.029 and HR 3.87, *P*=0.001, respectively) and, interestingly, their expression was highly correlated (*P*=0.002). Both TRKB and cmet proteins were significantly overexpressed in malignant STS compared with benign sarcomas (*P*=0.001 and 0.015, respectively) and TRKB overexpression was higher in cases with metastatic recurrence compared with local or no recurrence (*P*=0.05) (Supplementary Figure S4).

### Comparison of the prognostic power of tumour grade and gene expression score

The major prognostic determinants for localised extremity STS are histological subtype, grade and size, with grade alone being the most powerful single prognostic predictor. Five-year survival rates across all STS are 90, 70 and 45% for grades 1, 2 and 3, respectively (Guillou *et al*, 1997). To compare the prognostic power of gene expression with that of grade, we developed a scoring system based on expression of four promising genes (*RECQL4*, *FRRS1*, *CFH* and *MET*) and compared KM plots for metastasis-free survival (MFS) according to grade and score.

For scoring purposes, gene expression was classified as upregulated or downregulated according to expression above or below the level with highest sensitivity and specificity for metastasis detection within 3 years of follow-up, as determined by ROC analysis (which for the most part approximated the median value). In this dataset, upregulation of *RECQL4/MET* and downregulation of *CFH/FRRS1* favoured metastasis development. Assuming that each gene has equal weighting, the reference was set to represent the worst scenario for metastasis (i.e. upregulated *RECQL4* and *MET* and downregulated *CFH* and *FRRS1*). Scores between 0 and 4 were allocated according to the correspondence between observed and reference expression, with 4 representing the exact match/worst scenario score. Scores were then related to MFS using the log-rank test.

**Figure 8:**
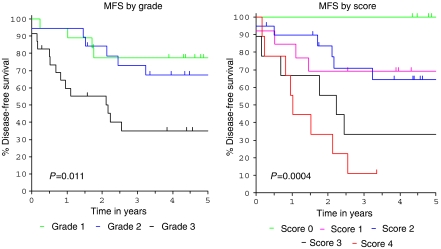
KM plots for metastasis-free survival (MFS) according to grade and gene score (see text for explanation of gene score): gene score is a more powerful prognostic predictor than grade in this dataset.

Interestingly, in this dataset, score was a more powerful predictor of MFS compared with grade (*P*-values 0.0004 and 0.011 respectively) (Figure 8), although these were not independent variables in a cox regression multivariate model. A score of 3 or 4 was associated with a much poorer MFS compared with scores of 2 or lower. Thus, even though the test value of gene expression is poor according to individual AUC values (0.68 each for *RECQL4* and *FRRS1*, 0.63 for *CFH*, 0.61 for *MET*, see Supplementary Figure S5), in this dataset the combined performance of these genes was a stronger predictor of MFS than grade, our current best prognostic predictor. These genes are therefore worthy of future exploration of their prognostic value in a larger cohort of extremity STS cases.

## DISCUSSION

In this study, we set out to assess the feasibility of using archival FFPET profiling for biomarker discovery in tumours where fresh-frozen tissue is in short supply. Using state-of-the-art technology at the time, including an RNA extraction kit from Ambion (Optimum, Ambion, TX, USA) and Affymetrix Plus 2.0 HG microarrays, we found that it is possible to identify promising clinically relevant molecules from archival FFPET, although further protocol amendments are needed to improve the sensitivity of this approach.

The reliable application of archival FFPET profiling to answer biologically relevant questions depends on the extent to which quantitative gene expression relationships are faithfully preserved in FFPET. As discussed earlier, several groups including ourselves have confirmed that archival FFPET profiles mirror those in paired FT samples (Bibikova *et al*, 2004; Haque *et al*, 2007), however it is not yet clear how close the resemblance should be to achieve this aim. In other words, does it matter that the sensitivity from FFPET is only 50% if the data are reliable (as evidenced by high specificity and positive predictive values of ∼95 and ∼92% respectively) and provide reproducible prognostic information? It is nevertheless desirable to improve the sensitivity of FFPET arrays. Low sensitivity rates of around 50% relative to FT arrays have been reported by others (Bibikova *et al*, 2004; Scicchitano *et al*, 2006) and may be due to the presence of residual nonreversed chemical modifications that reduce the efficiency or completely prevent *in vitro* transcription to cRNA (Godfrey *et al*, 2000; Bibikova *et al*, 2004; Lee *et al*, 2005b). Our archival FFPET RNA samples were similarly (and extensively) degraded, yet cRNA yields varied widely (5–28 *μ*g). This suggests that there was significant variation in inter-sample modification rates, presumably reflecting variation in routine processing conditions. However, despite the presence of variable ‘block effects’, relative quantification has been shown to be unaffected as long as data are normalised to an internal reference gene (Godfrey *et al*, 2000; Abrahamsen *et al*, 2003; Almeida *et al*, 2004), as indeed was performed for our samples. Furthermore, differential expression was validated by RT–PCR for approximately one-third of transcripts (i.e. fold change expression was similar by both assays). This is a promising result, especially considering that RT–PCR and microarray data from the same sample do not always correlate even when RNA quality is optimal. For example, RT–PCR comparability is lower for genes with low microarray signal intensities (weakly expressed or absent genes) – as demonstrated in the present study – and with increasing separation between the location of PCR primers and microarray probes (Tsien *et al*, 2001; Etienne *et al*, 2004; O’Sullivan *et al*, 2005; Wang *et al*, 2006). Thus RT–PCR confirmation of quantitative microarray data, even if only for a third of transcripts, lends further support to the hypothesis that expression patterns are preserved in archival FFPET, although further work is needed to test this fully, including evaluation of technical replicates in a larger dataset.

The use of oligo(dT) priming is a standard labelling approach, but may have contributed to low gene detection rates in FFPET as fragments discontinuous with the poly A tailed region cannot be anchored to oligo(dT) primers. Preferential chemical modification of adenine residues by formalin further compromises the suitability of oligo(dT) for FFPET substrates (Masuda *et al*, 1999; Paik *et al*, 2005). A direct comparison of random hexamer and oligo(dT) priming has confirmed that random primers give higher gene detection rates from FFPET (Xiang *et al*, 2003). The recently launched Nugen and Nugen FFPE protocols utilising both oligo(dT) and random priming are therefore especially promising for FFPET substrates (Nugen Technologies, CA, USA) and are likely to significantly improve the sensitivity of FFPET arrays.

The choice of microarray platform may also affect gene detection rates. Affymetrix Plus 2.0 arrays used in the present study contain multiple short (25-mer) probe sets located within ∼500 bp of the 3′ utr transcript regions. In our hands Plus 2.0 arrays are only marginally inferior to X3P arrays (by 3–5%, unpublished data), which contain probe sets within ∼300 bases of 3′utr and are marketed specifically for FFPET. The unique design features of newer platforms such as the Affymetrix Exon arrays may be better suited to archival FFPET interrogation and are worthy of further investigation of their ability to improve gene detection.

Finally, gene detection is likely to improve with the use of newer RNA extraction protocols that have been designed for superior reversal of chemical modifications, albeit that a proportion of these will remain irreversible (Masuda *et al*, 1999; Paik *et al*, 2005).

The second primary aim of this project was to establish whether archival FFPET microarray-selected transcripts contain prognostic relevance. Log-rank testing confirmed this to be the case for the 500- and 50-gene signatures and moreover functional annotation confirmed known cancer roles for a large number of identified genes. It would have been desirable to validate the full 50-gene signature but, as we did not have the means to test the performance of large numbers of genes by RT–PCR (including the full 50-gene signature), we selected twenty-four candidate genes for RT–PCR validation.

Successful RT–PCR validation was limited to some extent by the small training series used for prognostic modelling especially considering the heterogeneous histological nature of training samples although, arguably, histological heterogeneity is less important if the aim is to identify differentially regulated genes that drive tumour behaviour across all subtypes. In our dataset, survival analyses support prognostic roles for several differentially regulated transcripts, including *RECQL4*, *FRRS1*, *CFH*, *ADAMTS9*, *MNAB*, and to a lesser extent *MET*, *SOX4*, *NA[1562932_at]* and hypothetical protein *FLJ10292*. Interestingly, the combined prognostic effect of selected genes *RECQL4*, *FRRS1*, *CFH* and *MET*, as tested using a simple equal-weighted scoring system, was greater than that of tumour grade, our current best prognostic test. This finding alone strongly supports the hypothesis that archival FFPET microarray-selected genes contain valuable prognostic relevance.

We were particularly interested in the downstream prognostic effects of the protein tyrosine kinase family as the utility of targeting their proteins for treatment of specific cancers is well established and they are known to be highly expressed in a large proportion of bone and soft tissue sarcomas (Baird *et al*, 2005). We tested two protein tyrosine kinase receptors at protein level – cmet and TRKB. Dysregulation of their signalling pathways are known to be important mediators of the metastatic phenotype (Corso *et al*, 2005; Desmet and Peeper, 2006). These proteins were significantly upregulated in malignant compared to benign soft tissue tumours (implicating a role in tumorigenesis) and moreover significantly increased the risk of metastasis development in our samples. Cmet has previously been detected in 52–87% of STS (Fukuda *et al*, 1998; Kuhnen *et al*, 2003) and, an important proto-oncogene itself, also enables sarcoma cells to become hyper-responsive to HGF, a ubiquitously expressed effector of cell proliferation, motility and invasiveness (Cortner *et al*, 1995). Dysregulation of TRKB and its activating neurotrophin BDNF play a role in tumorigenesis and metastasis in a wide range of carcinomas (Han *et al*, 2007) but, to our knowledge, a role for TRKB in sarcoma tumorigenesis and metastasis development has not previously been described. Overexpression of TRKB also mediates cmet activation via upregulation of HGF (Hecht *et al*, 2005) and cooperation between TRKB and cmet has been implicated in the invasive capability of neuroblastoma cell lines. Evidence of highly correlated expression in our study suggests that similar cooperation between cmet and TRKB may be important for metastatic progression in STS and could represent dual targeting opportunities. Targeted therapies for cmet and TRKB are already under evaluation in other tumours (Christensen *et al*, 2005; Desmet and Peeper, 2006) and their potential value in STS warrants evaluation in future clinical trials.

In conclusion, our preliminary findings suggest that microarray gene detection from archival FFPET is reliable and capable of identifying candidate prognostic genes in human tumours using standard methodology, albeit with a gene detection rate of only ∼50% compared with frozen tissues. Despite the limitations of the approach we used, several candidate prognostic molecules for extremity STS were identified in this dataset and are worthy of prospective investigation of their prognostic value and/or potential as therapeutic targets in extremity STS.

We anticipate that the sensitivity of this approach will improve through the use of newer FFPET extraction protocols, combined oligo(dT) and random hexamer priming and newly-developed array platforms. The development of a highly reliable and robust protocol for microarray analysis of archival FFPET appears to be within reach and, with appropriate protocol improvements, promises to become a valuable research tool, providing an entry to the molecular investigation of tumours where supplies of FT are limited or nonexistent.

## Figures and Tables

**Table 1:** Candidate prognostic classifier genes for soft tissue sarcoma ranked by log_2_ discriminatory fold change

**Gene symbol**	**Affymetrix probe set id**	**Accession**	**Unigene**	**Log_2_^a^**	**Gene list source**	*P*-value*	**Cancer function**
PPP1R1A	205478_at	NM_006741	Hs.505662	3.28	List 2	0.001	Yes
NTRK2	221796_at	AA707199	Hs.494312	3.26	List 2	0.004	Yes
FRRS1	1570207_at	BC029438	Hs.454779	2.78	List 2	0.002	Not known
ADRB1	229309_at	AI625747	Hs.99913	2.76	List 2	0.001	Yes
CFH	213800_at	X04697	Hs.2637	−2.70	List 2	0.003	Yes
ZNF165	206683_at	NM_003447	Hs.55481	−2.58	List 2	0.006	Yes
CALCR	207887_s_at	AB022177	Hs.489127	2.50	List 2	0.002	Yes
CMAH	1554862_at	BC022302	Hs.484918	−2.49	List 2	0.006	Not known
MET	203510_at	BG170541	Hs.132966	−2.47	List 2	0.003	Yes
HMGA1	206074_s_at	NM_002131	Hs.518805	−2.43	List 2	0.005	Yes
PPP1R14A	227006_at	AA156998	Hs.348037	2.34	List 2	0.009	Yes
TYRP1	205694_at	NM_000550	Hs.270279	2.33	List 2	0.001	Yes
TPM4	235922_at	AW629304	Hs.466088	2.33	List 2	0.009	Not known
ADAMTS9	1556413_a_at	AF086538	Hs.549184	2.29	List 2	0.010	Yes
GHR	205498_at	NM_000163	Hs.125180	2.28	List 2	0.006	Yes
NA	1562932_at	BC015135	NA	2.05	List 2,3	0.001	Not known
NA	233162_at	AK024336	NA	1.91	List 2,3	0.004	Not known
SLC2A3	202499_s_at	NM_006931	Hs.419240	1.88	List 2,3	0.002	Yes
SOX4	213668_s_at	AI989477	Hs.357901	−1.87	List 3	b	Yes
CACNG1	206612_at	NM_000727	Hs.147989	1.59	List 2,3	0.004	Not known
RECQL4	213520_at	NM_004260	Hs.31442	−1.54	List 2,3	0.004	Yes
FLJ10292	218894_s_at	NM_018048	Hs.104650	−1.47	List 2,3	0.003	Not known
MNAB	231716_at	AF255304	Hs.533499	−1.45	List 3	b	Not known
MOXD1	209708_at	AY007239	Hs.6909	−1.45	List 2,3	0.003	Not known^b^

a Log_2_ fold changes differ from those obtained from scaled MAS5 processed data because unscaled data were used (see text).

^*^P-values obtained from List 2.

b Individual *P*-value not applicable as gene derived from List 3 where expression and significance of groups of genes were considered.
