# Acquisition of biologically relevant gene expression data by Affymetrix microarray analysis of archival formalin-fixed paraffin-embedded tumours

**DOI:** 10.1038/sj.bjc.6604506

**Published:** 2008-07-15

**Authors:** K M Linton, Y Hey, E Saunders, M Jeziorska, J Denton, C L Wilson, R Swindell, S Dibben, C J Miller, S D Pepper, J A Radford, A J Freemont

**Affiliations:** 1 Cancer Research UK Department of Medical Oncology, Christie Hospital NHS Foundation Trust, Wilmslow Road, Withington, Manchester, M20 4BX, UK; 2 School of Clinical and Laboratory Sciences, The University of Manchester, Oxford Road, Manchester, M13 9PT, UK; 3 Cancer Research UK Paterson Institute for Cancer Research, The University of Manchester, Wilmslow Road, Withington, Manchester, M20 4BX, UK; 4 Almac Diagnostics, Seagoe Industrial Estate, Craigavon, BT63 5QD, Northern Ireland; 5 School of Cancer and Imaging Sciences, The University of Manchester, Oxford Road, Manchester, M13 9PT, UK

**Correction to**: *British Journal of Cancer* (2008) **98**, 1403–1414; doi:10.1038/sj.bjc.6604316

During correction of this article, the affiliations of the authors were changed, therefore causing an error in the footnote attributing credit for the work involved in the paper.

The correct affiliations are shown above and the corrected footnote should read ‘This work is attributed to institutions 1–3 and 5 above’.

