# Supplementary material for: Acquisition of biologically relevant gene expression data by Affymetrix microarray analysis of archival formalin-fixed paraffin-embedded tumours
Source: Br J Cancer. 2008 Apr 1;98(8):1403–14. doi: 10.1038/sj.bjc.6604316 (PMC2361698; doi:10.1038/sj.bjc.6604316)
Supplement: Supplementary Information [file 6604316x1.doc]

**SUPPLEMENTARY INFORMATION for ‘Acquisition of biologically relevant gene expression data by Affymetrix microarray analysis of archival formalin fixed paraffin-embedded tumours’**

**A List of Supplementary Figures Page**

S1 Genorm average expression stability values of

six reference genes examined in 50 extremity soft

tissue sarcoma cases……………………………………………….. 2

S2 Comparison of qPCR data from paired frozen and

FFPET tissues……………………………………………………… 3

S3 Hazard ratios for metastasis and/or mortality from

extremity soft tissue sarcoma for selected candidate

genes and proteins…………………………………………………. 4

S4 Patterns of differential protein expression soft tissue

tumours according to benign or malignant types and

recurrence type…………………………………………………….. 5

S5 ROC curves and AUC values for four promising genes

included in the gene scoring system……………………………….. 6

**B List of Supplementary Tables**

S1 Affymetrix quality control results for FFPET and FT arrays……… 7

S2 Primer and probe details for RT-PCR detection of candidate genes in extremity soft tissue sarcoma……………………………………. 8

S3 Patient and tumour characteristics for 19 primary extremity array

training cases……………………………………………………….. 9

S4 Patient and tumour characteristics for 69 independent primary

extremity STS cases………………………………………………... 10

**C Gene Lists are given in the excel spreadsheets**

**A. Supplementary figures**

🡸 Least stable genes Most stable genes 🡺


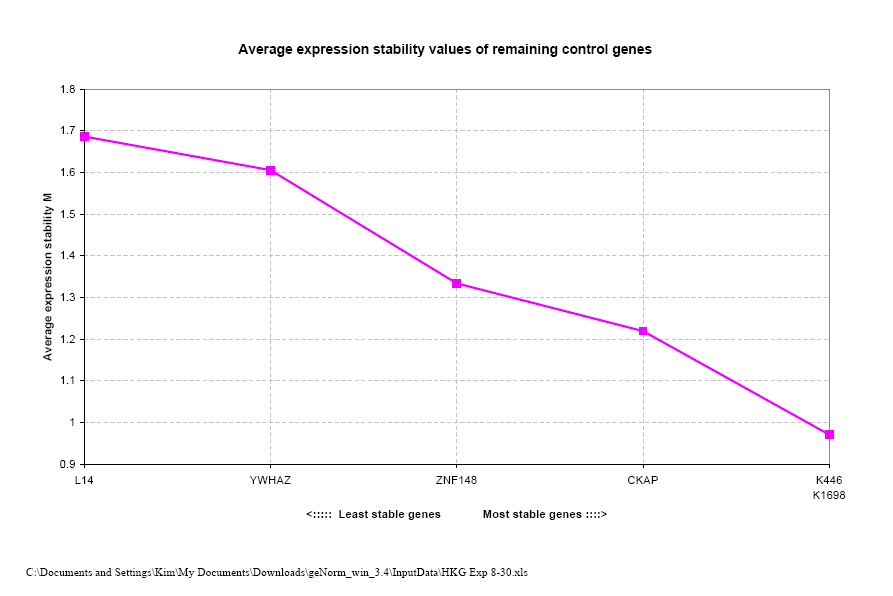


Average expression stability M

L14 YWHAZ ZNF148 CKAP INTS5

KIAA0446

**Figure S1. Genorm average expression stability values of six reference genes examined in 50 extremity soft tissue sarcoma cases.** Reference genes were selected from the most stable genes across arrays and their performance was examined by qPCR compared with commonly-used reference genes. The six genes shown here were the most stable of 15 tested genes (including GAPDH, ßactin, YWHAZ and L14); the two most stable genes - *KIAA0446* and *INTS5* – have the lowest M values and were used to normalise qPCR data. Genorm software is available online at: <http://medgen.ugent.be/~jvdesomp/genorm/>.

Sample 56 (KL35, FFPET)

Sample 56 (KL39, FT)

**Figure S2.** **Comparison of qPCR data from paired frozen and FFPET tissues:** Cts are on average 5-7 cycles later for transcript detection from FFPET than from FT, which for some transcripts results in non-detection of less abundant transcripts from FFPET (where Ct ≥ ~30 in FT)

Gene

Odds ratio (95% CI), p-value

5.16 (2.05, 12.8), p<0.000

0.64 (0.30, 1.39), p=0.264

0.49 (0.22, 1.09), p=0.079

2.25 (0.98, 5.21), p=0.057

0.45 (0.20, 1.02), p=0.055

2.09 (0.94, 4.63), p=0.069

0.44 (0.20, 0.97), p=0.041

0.35 (0.14, 0.83), p=0.017

2.09 (0.95, 4.61), p=0.068

0.48 (0.21, 1.09), p=0.080

RECQL4

NTRK2

FLJ10292

MNAB

ADAMTS9

MET

CFH

FRRS1

SOX4

1562932_at

Protein

Favours metastasis-free

survival

Favours metastasis ± mortality

TRKB (product of NTRK2)

3.87 (1.75, 8.58), p=0.001

2.37 (1.09, 5.14), p=0.029

0.1

0.5

0.2

1

2

5

10

cmet (product of MET)

**Figure S3.** **Hazard ratios for metastasis and/or mortality from extremity soft tissue sarcoma for selected candidate genes and proteins**

0

100

200

300

400

500

600

B

M

TRKB cMET

B

M

p=0.001

p=0.015

B = Benign M = Malignant

0

100

200

300

400

500

600

No

recurrence

Local

recurrence

Metastatic

recurrence

0

100

200

300

400

500

600

No

recurrence

Local

recurrence

Metastatic

recurrence

cMET

p=0.053

p=0.701

TRKB

**Figure S4. Patterns of differential protein expression soft tissue tumours according to benign or malignant types and recurrence type** (A) TRKB and c-met expression is significantly higher in malignant sarcoma, (B) A non-significant trend towards increased expression from no recurrence to metastatic recurrence is seen for TRKB, but no relationship between expression and recurrence is seen for c-met.

**Figure S5. ROC curves and AUC values for four promising genes included in the gene scoring system**

**B. Supplementary tables**

|  | Table S1 - Affymetrix quality control results for FFPET and FT arrays | | | | | | | | | | | | | | | |
| --- | --- | --- | --- | --- | --- | --- | --- | --- | --- | --- | --- | --- | --- | --- | --- | --- |
|  | **CEL file name**  **(sample ID in bold)** | | | **RIN** | | **260/**  **280** | **260/**  **230** | **total**  **RNA**  **(ng/µl)** | **cRNA**  **yield**  **(µg)** | **Scale**  **Factor** | **%**  **present** | | **GAPDH**  **3'/5'** | | **ß-actin**  **3'/5'** | |
|  | **Training FFPET cases** | | | | | | | | | | | | | | | |
|  | 0805_**KL1**_H_RD_3_2.CEL | | | 2.2 | | 2.0 | 2.1 | 1841 | 14 | 5.0 | 22.2 | | 28.0 | | 27.9 | |
|  | 0405_**KL2**_H_FL_4.CEL | | | 2.4 | | 2.0 | 2.0 | 847 | 17 | 6.6 | 22.1 | | 15.5 | | 49.5 | |
|  | 0705_**KL4**_H_FL_9.CEL | | | 2.3 | | 2.1 | 0.8 | 777 | 22 | 6.3 | 27.1 | | 34.8 | | 95.1 | |
|  | 0705_**KL5**_H_RD+L_11.CEL | | | 2.4 | | 2.1 | 2.1 | 1500 | 15 | 5.8 | 27.3 | | 61.3 | | 121.7 | |
|  | 0705_**KL6**_H_FL_12.CEL | | | 2.3 | | 2.0 | 1.7 | 447 | 15 | 7.6 | 24.5 | | 21.3 | | 53.8 | |
|  | 0705_**KL9**_H_RD_21.CEL | | | 2.1 | | 2.1 | 2.1 | 886 | 17 | 7.0 | 27.6 | | 96.9 | | 46.9 | |
|  | 0705_**KL10**_H_RD+L_22.CEL | | | 2.2 | | 2.0 | 2.0 | 484 | 22 | 6.9 | 26.3 | | 92.2 | | 128.1 | |
|  | 0705_**KL11**_H_RD_7b.CEL | | | 2.2 | | 2.0 | 1.9 | 1006 | 15 | 6.3 | 26.0 | | 119.5 | | 56.6 | |
|  | 0705_**KL12**_H_FL_13b.CEL | | | 2.3 | | 2.0 | 1.6 | 538 | 14 | 6.6 | 26.7 | | 78.3 | | 64.7 | |
|  | 0705_**KL14**_H_RD_29.CEL | | | 2.3 | | 2.0 | 1.9 | 1564 | 23 | 9.7 | 20.9 | | 24.7 | | 38.8 | |
|  | 0705_**KL15**_H_FL_30.CEL | | | 2.2 | | 2.1 | 1.8 | 930 | 15 | 8.2 | 24.4 | | 70.6 | | 130.6 | |
|  | 0705_**KL17**_H_FL_39.CEL | | | 2.3 | | 2.0 | 1.8 | 365 | 22 | 8.3 | 23.3 | | 17.4 | | 56.6 | |
|  | 0705_**KL19**_H_FL_46.CEL | | | 2.1 | | 2.0 | 2.0 | 498 | 15 | 6.6 | 26.0 | | 33.8 | | 36.9 | |
|  | 0705_**KL20**_H_RD+L_24b.CEL | | | 2.0 | | 2.1 | 1.5 | 1022 | 19 | 8.7 | 22.9 | | 28.5 | | 38.1 | |
|  | 0705_**KL25**_H_FL_41.CEL | | | 2.0 | | 2.0 | 1.7 | 141 | 20 | 8.8 | 23.4 | | 35.2 | | 42.9 | |
|  | 0705_**KL31**_H_RD+L_51.CEL | | | 2.4 | | 2.1 | 1.5 | 968 | 13 | 10.8 | 20.6 | | 126.1 | | 49.9 | |
|  | 0705_**KL35**_H_RD_56.CEL | | | * | | 2.1 | 1.1 | 1384 | 23 | 4.0 | 27.3 | | 54.8 | | 102.8 | |
|  | 0705_**KL36**_H_RD_58.CEL | | | 3.6 | | 2.1 | 0.5 | 710 | 16 | 5.4 | 27.5 | | 66.6 | | 135.6 | |
|  | 0805_**KL37**_H_FL_42_2.CEL | | | 2.3 | | 2.0 | 1.5 | 210 | 24 | 5.2 | 22.9 | | 17.7 | | 41.2 | |
|  | **Non-training FFPET cases** | | | | | | | | | | | | | | | |
|  | 0705_**KL3**_H_RD_8.CEL | | | 1.3 | | 1.9 | 1.9 | 1454 | 14 | 21.6 | 8.8 | | 1.7 | | 7.4 | |
|  | 0705_**KL7**_H_RL_14.CEL | | | 2.3 | | 2.0 | 2.0 | 1033 | 14 | 14.6 | 15.4 | | 16.5 | | 6.9 | |
|  | 0705_**KL8**_H_RD_17.CEL | | | 2.4 | | 1.9 | 1.8 | 533 | 15 | 14.3 | 15.8 | | 27.6 | | 37.0 | |
|  | 0405_**KL16**_H_FL_33.CEL | | | 2.2 | | 2.0 | 2.0 | 1487 | 28 | 3.6 | 32.3 | | 56.7 | | 202.0 | |
|  | 0705_**KL18**_H_RL_43.CEL | | | 2.3 | | 2.1 | 2.0 | 890 | 21 | 4.0 | 33.8 | | 84.9 | | 241.7 | |
|  | 0805_**KL21**_H_RD_31b_2.CEL | | | 2.2 | | 2.0 | 1.9 | 448 | 8 | 7.3 | 15.1 | | 14.6 | | 19.9 | |
|  | 0705_**KL22**_H_FL_35b.CEL | | | 2.2 | | 2.1 | 2.0 | 453 | 21 | 13.1 | 18.7 | | 507.5 | | 29.0 | |
|  | 0705_**KL23**_H_FL_37.CEL | | | 1.2 | | 2.0 | 1.4 | 176 | 11 | 17.6 | 11.7 | | 26.2 | | 51.4 | |
|  | 0705_**KL28**_H_RD_48.CEL | | | 2.3 | | 2.1 | 1.5 | 1139 | 18 | 13.2 | 16.9 | | 22.3 | | 12.7 | |
|  | 0705_**KL29**_H_FL_49X.CEL | | | 1.2 | | 2.0 | 1.8 | 287 | 17 | 13.1 | 16.7 | | 5.3 | | 16.6 | |
|  | 0705_**KL30**_H_RD_50.CEL | | | 2.2 | | 2.1 | 1.9 | 1065 | 25 | 12.4 | 18.5 | | 26.3 | | 16.8 | |
|  | 0705_**KL32**_H_RD_10c.CEL | | | 2.2 | | 2.0 | 2.0 | 1182 | 17 | 14.7 | 14.9 | | 6.4 | | 6.7 | |
|  | 0705_**KL33**_H_RD_52.CEL | | | 2.3 | | 2.0 | 1.7 | 430 | 14 | 13.7 | 15.2 | | 371.3 | | 18.4 | |
|  | 0705_**KL34**_H_RD_54.CEL | | | 2.4 | | 2.1 | 1.9 | 822 | 15 | 10.4 | 18.8 | | 67.8 | | 70.2 | |
|  | 0805_**KL38**_H_RD_44_2.CEL | | | 2.3 | | 2.0 | 1.2 | 298 | 12 | 9.3 | 12.0 | | 6.5 | | 12.5 | |
|  | **KL24** | | |  | | 2.0 | 1.6 | 245 |  | insufficient total RNA – not hybridised | | | | | | |
|  | **KL26** | | |  | | 2.0 | 1.6 | 290 |  | insufficient total RNA - 2^nd^ prep (KL37)  labeled, pooled and hybridised | | | | | | |
|  | **KL27** | | |  | | 2.0 | 0.8 | 208 | 8 | insufficient total RNA - 2^nd^ prep (KL38)  labeled, pooled and hybridised | | | | | | |
|  | **Control FT cases** | | | | | | | | | | | | | | | |
|  | 0705_**KL39**_H_RD_56F.CEL  (paired with KL35) | | | 5.5 | | 2.1 | 1.4 | 313 | 122 | 0.8 | 48.9 | | 1.4 | | 3.3 | |
|  | 0705_**KL40**_H_RD_58F.CEL  (paired with KL36) | | | 3.1 | | 2.1 | 1.1 | 418 | 92 | 1.0 | 53.5 | | 1.6 | | 1.9 | |
| **Table S2 - Primer and probe details for RT-PCR detection of candidate genes in extremity soft tissue**  **sarcoma** | | | | | | | | | | | | | | | |  |
| **Gene**  **Symbol** | | **Affymetrix**  **probeset**  **ID** | **Probe-**  **Library**  **Probe^‡^** | | **Forward Primer*** | | | | **Reverse Primer*** | | | **amplicon**  **length**  **(nt)** | | **Array &**  **qPCR**  **targets** | |  |
| **Candidate genes of interest** | | | | | | | | | | | | | | | |  |
| SLC2A3 | | 202499_s_at | 36 | | ggtgaccttgcaacttcatgt | | | | aaatgggaccctgccttact | | | 73 | | Identical | |  |
| CALCR | | 207887_s_at | 3 | | gaaggccaccatgatcctt | | | | ctccagggaaagacgacaaa | | | 60 | | Identical | |  |
| GHR | | 205498_at | 4 | | gaagcaagcttaatggctgataa | | | | catgttgaactttaaacactcaattct | | | 90 | | Identical | |  |
| CMAH | | 1554862_at | 6 | | aactgctgttctttcaaaataccc | | | | tcagttgtttgttcgatgctg | | | 71 | | Non-  identical | |  |
| PPP1R14A | | 227006_at | 17 | | gacgtggagaagtggatcg | | | | ttgatctcatcgggcatgt | | | 77 | | Identical | |  |
| MNAB | | 231716_at | 25 | | cagagtttaggtgaagaccatgtg | | | | gcaagaagttaccggcagaa | | | 66 | | Identical | |  |
| TYRP1 | | 205694_at | 34 | | tattgcctgtgtttgccact | | | | aggtagttcctttttcagcgaat | | | 61 | | Identical | |  |
| FRRS1 | | 1570207_at | 47 | | cctaagaaatttgcagtaaagaaaca | | | | tggctcacagtgacactcaat | | | 99 | | Identical | |  |
| ZNF165 | | 206683_at | 47 | | tgagctcacatcttattcgacac | | | | ctcttccacactcactgcattc | | | 75 | | Identical | |  |
| MET | | 203510_at | 48 | | accaaaatggctacacactgg | | | | cattcaatgggatcttcgtga | | | 60 | | Non-  identical | |  |
| PPP1R1A | | 205478_at | 56 | | cccagacacagaagtggagtc | | | | gggatgcattctgcagtttt | | | 69 | | Identical | |  |
| RECQL4 | | 213520_at | 61 | | tcagaggctagggcagtgac | | | | ctcgttcccacaccctgt | | | 61 | | Identical | |  |
| CACNG1 | | 206612_at | 62 | | cacagttgggggagcaga | | | | agctccaggggaagttgg | | | 66 | | Identical | |  |
| ADAMTS9 | | 1556413_a_at | 65 | | gacaccaggacacagttaattcag | | | | gccagacaggcagagaaagt | | | 61 | | Identical | |  |
| MOXD1 | | 209708_at | 71 | | tgatcataggctgaggagaaaaa | | | | ataagtcacctcacagcactgg | | | 63 | | Identical | |  |
| TPM4 | | 235922_at | 72 | | tggttgcagagtgtttttgc | | | | actcctcctaccaccttcagtaac | | | 63 | | Identical | |  |
| FLJ10292 | | 218894_s_at | 76 | | acacgttggctgcgtttt | | | | ccctacgtagtagcgcaggta | | | 83 | | Non-  identical | |  |
| CFH | | 213800_at | 78 | | aacttctgatcgaaggtcatcc | | | | ttggcccttgtcatctttg | | | 60 | | Identical | |  |
| SOX4 | | 213668_s_at | 85 | | gcagcttcagttcgtcgtc | | | | gcagtagtccgggaactcg | | | 92 | | Identical | |  |
| NA | | 1562932_at | 14 | | aagctctgttttggatttatcctg | | | | aaagaaagcctggagcacaa | | | 110 | | Non-  identical | |  |
| ADRB1 | | 229309_at | 24 | | gaagaaggcagccattgatt | | | | catcgctactgggactgaca | | | 67 | | Non-  identical | |  |
| NRTK2 | | 221796_at | 34 | | ttcatgttcaaccatttgctg | | | | aaaaacagggcatgagaatga | | | 64 | | Non-  identical | |  |
| NA | | 233162_at | 68 | | ttgctggctggcttcttc | | | | ccagcagacggagttttgat | | | 74 | | Non-  identical | |  |
| HMGA1 | | 206074_s_at | 68 | | catcccagccatcactcttc | | | | gagctcgactcactcatcttcc | | | 61 | | Non-  identical | |  |
| **Reference (housekeeping) genes** | | | | | | | | | | | | | | | |  |
| KIAA0446 | | 32091_at | 14 | | tggagttttggtcggtaatca | | | | agtgctccaatcaggtttcc | | | 67 | | Identical | |  |
| ZNF148 | | 203318_s_at | 33 | | cagaatttcccttggtgaatg | | | | tgtggcatcaggtgaagatg | | | 65 | | Identical | |  |
| INTS5 | | 53968_at | 76 | | agggagggtggaggtgag | | | | gttgcggtggaggacact | | | 63 | | Identical | |  |
| nt = nucleotides (amplicon length)  ^‡^ Labeled probes from Human Universal ProbeLibrary (Roche, Switzerland), * Primers from Invitrogen (Paisley, UK) | | | | | | | | | | | | | | | |  |

| **Table S3 – Patient and tumour characteristics for 19 primary extremity array training cases** | | | | | | | |
| --- | --- | --- | --- | --- | --- | --- | --- |
| **Array name**  **(sample ID in bold)** | **Sex, age** | **Diagnosis** | **Grade*** | **Tumour size (mm)** | **Metastatic outcome** | **DRFS (months)** | **Mortality**  **status^‡^** |
| **FFPET samples used for prognostic modelling** | | | | | | | |
| 0805_**KL1**_H_RD_3_2 | M, 87 | LS-M/P | 3 | 120 | yes | 29 | alive |
| 0405_**KL2**_H_FL_4 | M, 70 | LMS | 3 | 160 | no | 53 | alive |
| 0705_**KL4**_H_FL_9 | M, 86 | LS-S | 2 | 25 | no | 81 | alive |
| 0705_**KL5**_H_RD+L_11 | F, 86 | LMS | 2 | 60 | yes | 46 | dead |
| 0705_**KL6**_H_FL_12 | F, 56 | LS-M | 1 | 50 | no | 74 | alive |
| 0705_**KL9**_H_RD_21 | F, 66 | SS-B | 3 | 120 | yes | 19 | alive |
| 0705_**KL10**_H_RD+L_22 | F, 85 | LMS | 3 | 200 | yes | 5 | dead |
| 0705_**KL11**_H_RD_7b | M, 91 | LMS | 3 | 80 | yes | 9 | dead |
| 0705_**KL12**_H_FL_13b | M, 50 | LS-M/RC | 3 | 120 | no | 80 | alive |
| 0705_**KL14**_H_RD_29 | F, 49 | SS | 3 | 85 | yes | 23 | alive |
| 0705_**KL15**_H_FL_30 | M, 98 | LMS | 1 | 90 | no | 21 | dead |
| 0705_**KL17**_H_FL_39 | M, 61 | SS-B | 1 | 30 | no | 76 | alive |
| 0705_**KL19**_H_FL_46 | M, 56 | LS-M/RC | 2 | 65 | no | 78 | alive |
| 0705_**KL20**_H_RD+L_24b | F, 85 | SS-MO | 3 | 130 | yes | 23 | dead |
| 0705_**KL25**_H_FL_41 | M, 58 | LS-M/RC | 2 | 170 | no | 52 | alive |
| 0705_**KL31**_H_RD+L_51 | M, 75 | LMS | 2 | 60 | yes | 2 | dead |
| 0705_**KL35**_H_RD_56 | M, 70 | SPINDLE-P | 3 | 120 | yes | 6 | dead |
| 0705_**KL36**_H_RD_58 | F, 54 | LMS | 2 | 130 | yes | 34 | alive |
| 0805_**KL37**_H_FL_42_2 | F, 65 | LS-D | 3 | 150 | no | 47 | alive |
| *graded according to FNCLCC/Trojani 3-grade system; ^‡^at the time of analysis; M=male; F=female ; LS=liposarcoma; LMS=leiomyosarcoma; SS=synovial sarcoma; B=biphasic; C=cutaneous; D=dedifferentiated;M=myxoid; MO=monophasic; P=pleomorphic; RC=round cell; S=spindle; DRFS = distant recurrence-free survival, defined as date of diagnosis to date of metastasis (metastatic outcome) or date of diagnosis to date of last follow-up (non-metastatic outcome) | | | | | | | |

| Table S4 – Patient and tumour characteristics for 69 independent primary extremity STS cases | | | | | | | | | |
| --- | --- | --- | --- | --- | --- | --- | --- | --- | --- |
| **Sample ID**  **(array ID)** | **Sex** | **Age** | **Diagnostic**  **Subgroup** | **Grade** | **Size (mm)** | **Recurrence** | **DRFS (months)** | **Status^‡^** | **Exp(s)** |
| **Leiomyosarcomas (n=23)** | | | | | | | | | |
| 17 (KL8) | M | 77 | * | 3 | 75 | distant | 19 | dead | AN,Q^X^,I |
| 19 | F | 56 | * | 3 | 75 | distant | 17 | dead | Q,I |
| 33 (KL16) | M | 46 | * | 2 | 20 | none | 48 | alive | AN,Q,I |
| 36 | M | 67 | * | 3 | 115 | none | 54 | alive | Q,I |
| 37 (KL23) | M | 61 | * | 2 | 100 | none | 68 | alive | AN,Q,I |
| 40 | F | 78 | M/P | 1 | 50 | none | 102 | alive | Q,I |
| 44 (KL38) | M | 66 | * | 3 | 35 | distant | 30 | dead | AN,Q,I |
| 52 (KL33) | M | 83 | P | 2 | 40 | distant | 33 | dead | AN,Q,I |
| 81 | M | 82 | * | 1 | * | distant | 110 | dead | Q,I |
| 85 | F | 70 | * | 2 | 60 | distant | 26 | dead | I |
| 86 | F | 89 | * | 3 | 50 | distant | 19 | dead | Q^X^,I |
| 88 | F | 73 | * | 2 | 60 | local | 96 | alive | Q^X^,I |
| 91 | F | 91 | P | 3 | 195 | distant | 4 | dead | Q,I |
| 92 | M | 82 | * | 3 | 220 | none | 3 | dead | Qx,I |
| 93 | F | 90 | * | 3 | 110 | distant | 11 | dead | Q,I |
| 94 | M | 60 | * | 3 | 170 | distant | 37 | dead | Q,I |
| 96 | F | 78 | * | 2 | 80 | local | 115 | alive | Q^X^,I |
| 101 | F | 54 | * | 3 | 110 | distant | 13 | dead | Q,I |
| 102 | F | 87 | * | 3 | 270 | distant | 30 | dead | Q,I |
| 115 | M | 80 | * | 2 | 60 | distant | 5 | dead | I |
| 116 | M | 54 | P | 3 | 75 | none | 66 | alive | I |
| 122 | F | 83 | C | 2 | 15 | local | 56 | alive | I |
| 123 | M | 79 | * | 3 | 40 | none | 59 | dead | I |
| **Liposarcomas (n=32)** | | | | | | | | | |
| 1 | M | 82 | WD | 1 | 210 | local | 73 | alive | Q,I |
| 2 | F | 78 | WD | 1 | 150 | none | 46 | alive | Q,I |
| 5 | M | 51 | WD | 1 | 215 | none | 52 | alive | Q,I |
| 6 | F | 72 | M/RC | 1 | 70 | none | 105 | alive | I |
| 8 (KL3) | M | 42 | M/RC | 2 | 150 | distant | 52 | dead | AN,Q^X^,I |
| 15 | F | 80 | * | 1 | 24 | local | 68 | alive | Q,I |
| 16 | M | 81 | M/RC | 2 | 90 | local | 67 | alive | Q^X^,I |
| 18 | M | 74 | M/P | 2 | 30 | local | 81 | alive | Q,I |
| 20 | M | 50 | M | 1 | 90 | none | 59 | alive | Q,I |
| 26 | M | 79 | M/RC | 1 | * | distant | 28 | dead | Q,I |
| 27 | F | 63 | M | 1 | 80 | none | 57 | alive | Q,I |
| 31b (KL21) | M | 67 | P | 3 | 100 | distant | 28 | dead | AN,Q,I |
| 32 | F | 44 | M/RC | 3 | 145 | none | 107 | alive | Q^X^,I |
| 34 | M | 65 | M/RC | 2 | 240 | none | 53 | alive | Q,I |
| 35b (KL22) | M | 51 | M | 3 | 160 | none | 82 | alive | AN,Q,I |
| **(continued)** | | | | | | | | | |
| **Table S4 continued** | | | | | | | | | |
| **Sample ID**  **(array ID)** | **Sex** | **Age** | **Diagnostic**  **Subgroup** | **Grade** | **Size (mm)** | **Recurrence** | **DRFS (months)** | **Status^‡^** | **Exp(s)** |
| 38 | F | 53 | WD | 1 | 28 | none | 65 | alive | Q,I |
| 43 (KL18) | F | 88 | P | 3 | 80 | none | 62 | alive | AN,Q,I |
| 47 | M | 61 | WD | 1 | 60 | local | 140 | alive | Q^X^,I |
| 48 (KL28) | F | 51 | M/RC | 2 | 160 | distant | 31 | dead | AN,Q,I |
| 50 (KL30) | F | 44 | M/WD | 2 | 140 | distant | 18 | alive | AN,Q,I |
| 53 | M | 67 | M | 2 | 155 | none | 153 | alive | Q,I |
| 89 | M | 53 | M | 2 | 80 | none | 40 | alive | Q,I |
| 90 | M | 50 | M/RC | 1 | 330 | distant | 43 | dead | Q^X^,I |
| 106 | F | 76 | M | 2 | 140 | loc & distant | 63 | dead | I |
| 107 | M | 57 | P | 1 | 16 | none | 202 | alive | I |
| 108 | M | 65 | P | 3 | 70 | none | 32 | dead | I |
| 109 | F | 84 | M/P | 3 | 140 | none | 51 | alive | I |
| 110 | F | 85 | M | 1 | 170 | none | 159 | alive | I |
| 111 | F | 75 | * | 2 | 160 | none | 109 | alive | I |
| 112 | M | 70 | D | 2 | 220 | none | 71 | alive | I |
| 113 | M | 83 | WD | 1 | * | * | 92 | dead | I |
| 114 | M | 50 | P | 2 | 110 | distant | 74 | dead | I |
| **Synovial Sarcomas (n=14)** | | | | | | | | | |
| 10c (KL32) | M | 60 | B | 1 | 90 | distant | 19 | dead | AN,Q,I |
| 14 (KL7) | F | 43 | MO | 2 | 50 | local | 86 | alive | AN,Q,I |
| 23 | M | 48 | P | 1 | 48 | none | 88 | alive | Q,I |
| 49x (KL29) | M | 49 | * | 1 | 10 | none | 55 | alive | AN,Q,I |
| 54 (KL34) | M | 35 | B | 1 | * | distant | 8 | dead | AN,Q,I |
| 55 | M | 45 | B | 2 | 25 | distant | 132 | dead | Q |
| 64 | M | 43 | B | 2 | 30 | distant | 27 | dead | Q,I |
| 80 | M | 51 | MO | 1 | 40 | loc & distant | 30 | dead | Q,I |
| 95 | F | 66 | B | 3 | 70 | distant | 19 | dead | Q^X^,I |
| 103 | F | 39 | MO | 2 | 70 | none | 91 | alive | Q,I |
| 117 | M | 40 | * | 1 | 35 | none | 68 | alive | I |
| 118 | F | 41 | MO | 1 | 9 | none | 45 | alive | I |
| 119 | F | 41 | MO | 1 | 30 | none | 59 | alive | I |
| 120 | F | 92 | * | 2 | 250 | * | 2 | dead | I |
| **AN = array non-training case; I = IHC case; Q = qPCR case, ^x^ = case excluded from analysis**  *not otherwise specified/not known; ^‡^at the time of analysis; M=male; F=female; B=biphasic; C=cutaneous; D=dedifferentiated;M=myxoid; MO=monophasic; P=pleomorphic; RC=round cell; S=spindle; WD=well differentiated; DRFS=distant recurrence free survival (time in months from diagnosis to metastatic recurrence or last follow-up (for cases with no distant recurrence) | | | | | | | | | |
